# Identification of Genes Linking Natural Killer Cells to Apoptosis in Acute Myocardial Infarction and Ischemic Stroke

**DOI:** 10.3389/fimmu.2022.817377

**Published:** 2022-04-01

**Authors:** Lele Feng, Ruofei Tian, Xingdou Mu, Cheng Chen, Yuxi Zhang, Jun Cui, Yujie Song, Yingying Liu, Miao Zhang, Lei Shi, Yang Sun, Ling Li, Wei Yi

**Affiliations:** ^1^ Department of Cardiovascular Surgery, Xijing Hospital, The Fourth Military Medical University, Xi’an, China; ^2^ National Translational Science Center for Molecular Medicine and Department of Cell Biology, Fourth Military Medical University, Xi’an, China; ^3^ Department of Breast and Thyroid Surgery, Xijing Hospital, Fourth Military Medical University, Xi’an, China; ^4^ Department of Geriatrics, Xijing Hospital, The Fourth Military Medical University, Xi’an, China; ^5^ Department of Internal Medicine, Central Health Center of Huilong Town, Shangluo, China; ^6^ College of Life Science, Northwest University, Xi’an, China; ^7^ The Second Clinical Medicine College, Shaanxi University of Chinese Medicine, Xianyang, China

**Keywords:** natural killer cell, apoptosis, myocardial infarction, ischemic stroke, gene expression

## Abstract

Natural killer (NK) cells are a type of innate lymphoid cell that are involved in the progression of acute myocardial infarction and ischemic stroke. Although multiple forms of programmed cell death are known to play important roles in these diseases, the correlation between NK cells and apoptosis-related genes during acute myocardial infarction and ischemic stroke remains unclear. In this study, we explored the distinct patterns of NK cell infiltration and apoptosis during the pathological progression of acute myocardial infarction and ischemic stroke using mRNA expression microarrays from the Gene Expression Omnibus database. Since the abundance of NK cells correlated positively with apoptosis in both diseases, we further examined the correlation between NK cell abundance and the expression of apoptosis-related genes. Interestingly, APAF1 and IRAK3 expression correlated negatively with NK cell abundance in both acute myocardial infarction and ischemic stroke, whereas ATM, CAPN1, IL1B, IL1R1, PRKACA, PRKACB, and TNFRSF1A correlated negatively with NK cell abundance in acute myocardial infarction. Together, these findings suggest that these apoptosis-related genes may play important roles in the mechanisms underlying the patterns of NK cell abundance and apoptosis in acute myocardial infarction and ischemic stroke. Our study, therefore, provides novel insights for the further elucidation of the pathogenic mechanism of ischemic injury in both the heart and the brain, as well as potential useful therapeutic targets.

## Introduction

Cardiovascular and cerebrovascular diseases are associated with high mobility and mortality and are the leading causes of death worldwide (1). It was recently reported that cardiovascular diseases (CVDs), which include coronary heart disease, heart failure, stroke, and hypertension, have a prevalence of 49.2% in adults which increases with age in both males and females (2). It is therefore highly important to study the mechanisms underlying CVDs in order to develop improved methods of treatment and prevention.

Both acute myocardial infarction (AMI) and ischemic stroke (IS) are characterized by ischemia and hypoxia in the target organ, as well as inflammation and multiple forms of cell death. Immune cells play important roles in the occurrence, progression, and outcome of CVDs (3, 4). In particular, studies have reported that natural killer (NK) cells are crucially involved in AMI and IS (5, 6). NK cells are type I innate lymphoid cells that exhibit a lymphoid cellular morphology without antigen specificity, express the transcription factor T-bet, and produce IFN-γ, perforin, and granzyme B (7). Patients with coronary artery disease showed a significant reduction in the number of circulating NK cells compared with healthy controls (4, 8, 9). In IS, NK cells display distinct patterns of infiltration characterized by an increased accumulation in the brain and decreased accumulation in the peripheral blood (5). However, few studies have compared the differences in immune cell infiltration during AMI and IS. Furthermore, the role of NK cells in AMI and IS remains poorly characterized.

Since the mid-1980s, when apoptosis was the only well-defined form of regulated cell death, more than 10 other mechanistically distinct forms of programmed cell death have been recognized (10). Most recently, Gasdermin D(GSDMD)-mediated cardiomyocyte pyroptosis was reported to contribute toward heart damage during myocardial infarction and reperfusion injury (11). Although various therapies targeting these different forms of cell death have produced favorable results in preclinical studies and can effectively reduce adverse remodeling after AMI (12, 13), the correlations between immune cell infiltration and programmed cell death in AMI and IS and their pathological mechanisms remain unclear.

In this study, we performed bioinformatics analyses to compare the patterns of immune cell infiltration and cell death in patients with AMI or IS at the transcriptional level. Moreover, we identified correlations between NK cell infiltration and the expression of specific apoptosis-related genes. Together, the novel analyses performed in this study may provide new avenues for the study of AMI and IS, as well as the development of beneficial clinical treatments.

## Materials and Methods

### Data Collection and Processing

**Figure 1:**
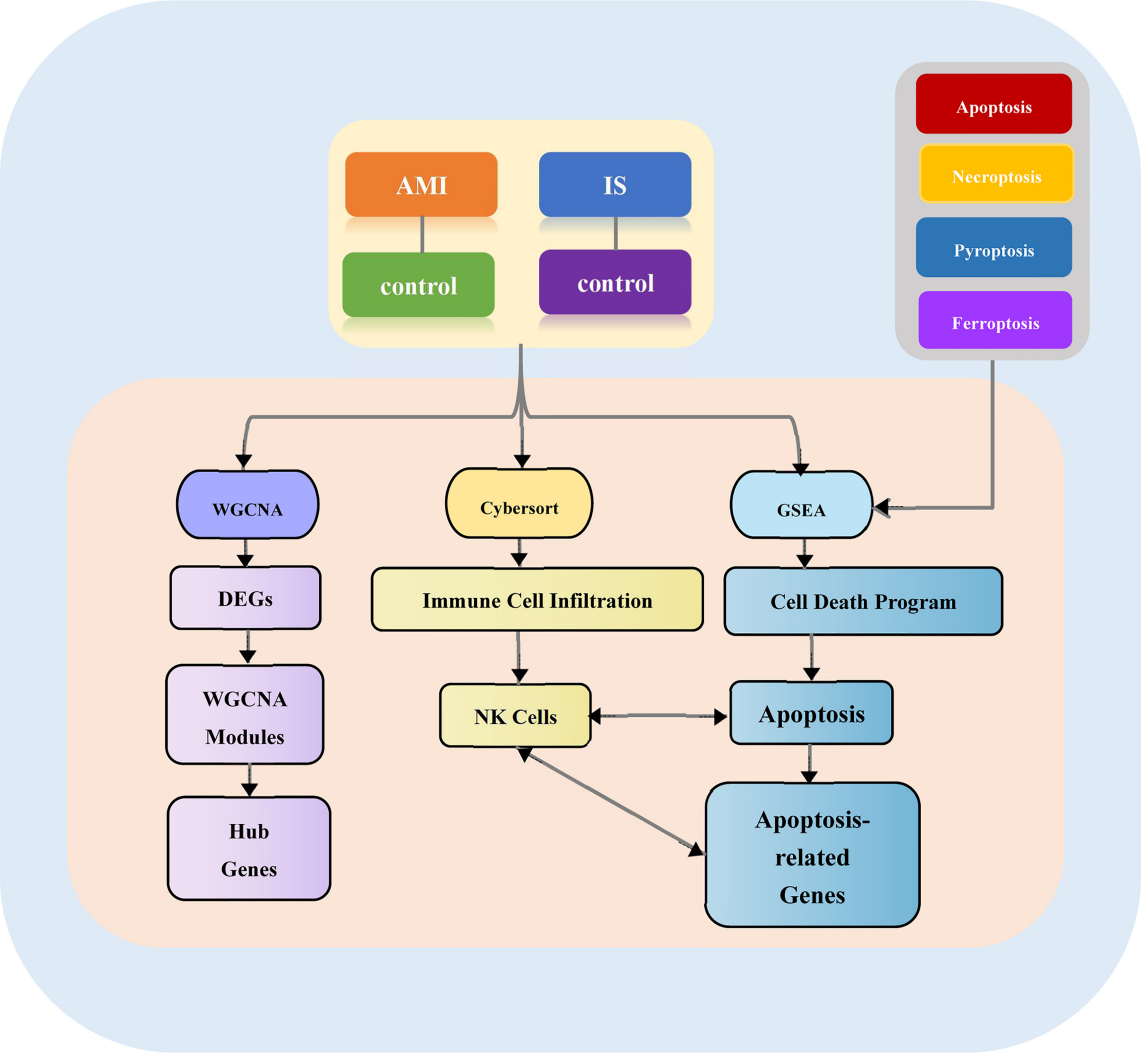
Study flowchart. AMI, acute myocardial infarction; DEGs, differentially expressed genes; WGCNA, weighted gene co-expression network analysis; GSEA, gene set enrichment analysis; NK cells, natural killer cells.

The overall design of our study is illustrated in **Figure 1**. First, we acquired the gene expression profiles of patients with AMI and IS from the Gene Expression Omnibus (GEO) database (http://www.ncbi.nlm.nih.gov/geo). Five mRNA microarray datasets were downloaded: GSE59867, GSE22255, GSE58294, GSE48060, and GSE16561. These datasets met the following criteria: (1) data acquired using microarray platforms detecting >15,000 genes; (2) ≥ 40 patients recruited; (3) all samples from *Homo sapiens*.

GSE59867 was based on the Affymetrix Human Gene 1.0 ST Array [transcript (gene) version]; 111 AMI and 46 control samples derived from peripheral blood mononuclear cells (PBMCs) were analyzed. GSE22255, GSE58294, and GSE48060 were based on Affymetrix Human Genome U133 Plus 2.0 Arrays. For GSE22255, gene expression profiling was performed on PBMCs from 20 patients with IS and 20 sex-and age-matched controls. For GSE58294, 23 control samples and 69 cardioembolic stroke samples were assayed. For GSE48060, blood samples from 31 patients with AMI and 21 controls were analyzed. GSE16561 was based on the Illumina HumanRef-8 v3.0 expression beadchip analysis of total RNA extracted from the whole blood of 39 patients with IS and 24 healthy controls. GSE22255, GSE59867, and GSE58294 were used as basic datasets for screening differentially expressed genes (DEGs), immune cell infiltration analysis, and functional enrichment analysis. GSE48060 and GSE16561 were used as the validation cohorts. The details of these datasets are provided in **Table 1**.

**Table 1:** GEO dataset information.

GEO accession ID	Disease	Platform	Samples (total no.)	No. of cases	No. \of controls	Country	Reference
**Basic analysis datasets**							
GSE59867	Acute myocardial infarction	GPL6244	Peripheral blood mononuclear cells (157)	111	46	Poland	(14)
GSE22255	Ischemic stroke	GPL570	Peripheral blood mononuclear cells (40)	20	20	Portugal	(15)
GSE58294	Ischemic stroke	GPL570	Whole blood samples (92)	69	23	USA	(16)
**Validation datasets**							
GSE48060	Acute myocardial infarction	GPL570	Whole blood samples (52)	31	21	USA	(17)
GSE16561	Ischemic stroke	GPL6883	Whole blood samples (63)	39	24	USA	(18)

### Data Merging

The GEO datasets were merged using the “sva” R package (19). GSE22255 and GSE58294 were merged as the input data for DEG analysis, whereas GSE22255, GSE58294, andGSE59867 were merged as the input data for weighted gene co-expression network analysis (WGCNA) and to evaluate immune cell infiltration.

### DEG Screening

DEG analysis was performed on the GEO array data using the “Limma” R package (20). Genes with a *P*-value < 0.05 and |log2fold change (FC)| > log2(1.2) were considered DEGs.

### Functional Enrichment Analysis

Gene ontology (GO) analysis, Kyoto encyclopedia of genes and genomes (KEGG) analysis, and gene set enrichment analysis (GSEA) (21) were performed using the “clusterProfiler” (22) and “GSEABase” R packages.

### WGCNA

WGCNA was performed using the “WGCNA” (23) R package with the top 5000 DEGs as input genes. Hub genes in the WGCNA modules were identified using Cytoscape (24).

### Evaluation of Immune Cell Infiltration

The infiltration of 22 immune cell types in the AMI and IS samples used in this study was analyzed using CIBERSORT (25). The expression data were imported into CIBERSORT using R and then iterated 1000 times to estimate the relative proportion of each immune cell type.

### GSEA

GSEA was performed to find enriched terms in four cell death pathways. Differences were considered statistically significant if *p* < 0.05 or FDR (false discovery rate)  < 0.25 and |NES| >1.

### Gene Set Variation Analysis (GSVA)

GSVA (26) was performed to transform the GEO gene expression matrices into programmed cell death enrichment matrices to detect subtle pathway activity changes over a sample population. And the results were used to analyse the relationship between immune infiltration and the cell death.

## Results

### Identifying DEGs in AMI and IS

**Figure 2:**
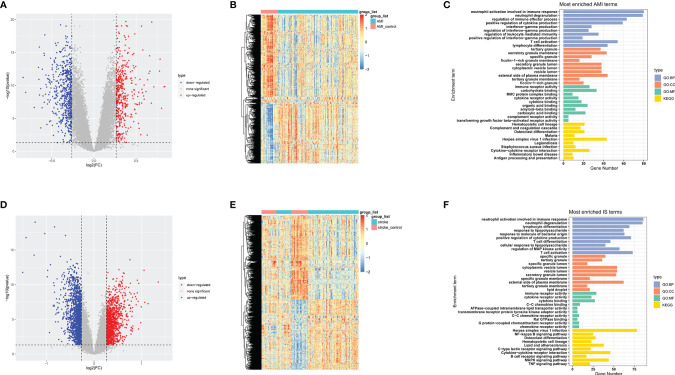
Data preprocessing, DEG identification, and enrichment analyses. **(A)** Volcano plots of DEGs in AMI. **(B)** Heatmaps of DEGs in AMI. **(C)** GO and KEGG enrichment analyses in AMI. **(D)** Volcano plots of DEGs in IS. **(E)** Heatmaps of DEGs in IS. **(F)** GO and KEGG enrichment analysis in IS. Statistical significance was determined by hypergeometric distribution analysis and the *P*-values were calculated by Fisher’s exact test. CC, cell component; BP, biological process; MF, molecular function.

To identify DEGs in the basic datasets obtained from the GEO, we first merged the expression matrices to eliminate between-batch differences. For AMI, 1002 DEGs were obtained, 429 of which were significantly upregulated and 573 of which were downregulated. Meanwhile, 2488 DEGs were obtained for IS, of which 1133 were significantly upregulated and 1355 were significantly downregulated. Volcano plots and heatmaps of these DEGs are shown in **Figures 2A, B, D, E**. To further investigate the functions of these DEGs, we conducted GO and KEGG enrichment analyses. The top 10 enriched terms are listed (**Figures 2C, F**). Notably, the DEGs in both AMI and IS were mainly associated with immune system activation, immune cell activities, inflammatory cytokine release, and inflammation-related pathways. Together, these findings suggest that the DEGs are likely to be associated with inflammatory processes (**Figures 2C, F**).

### WGCNA and Identification of Key Modules

**Figure 3:**
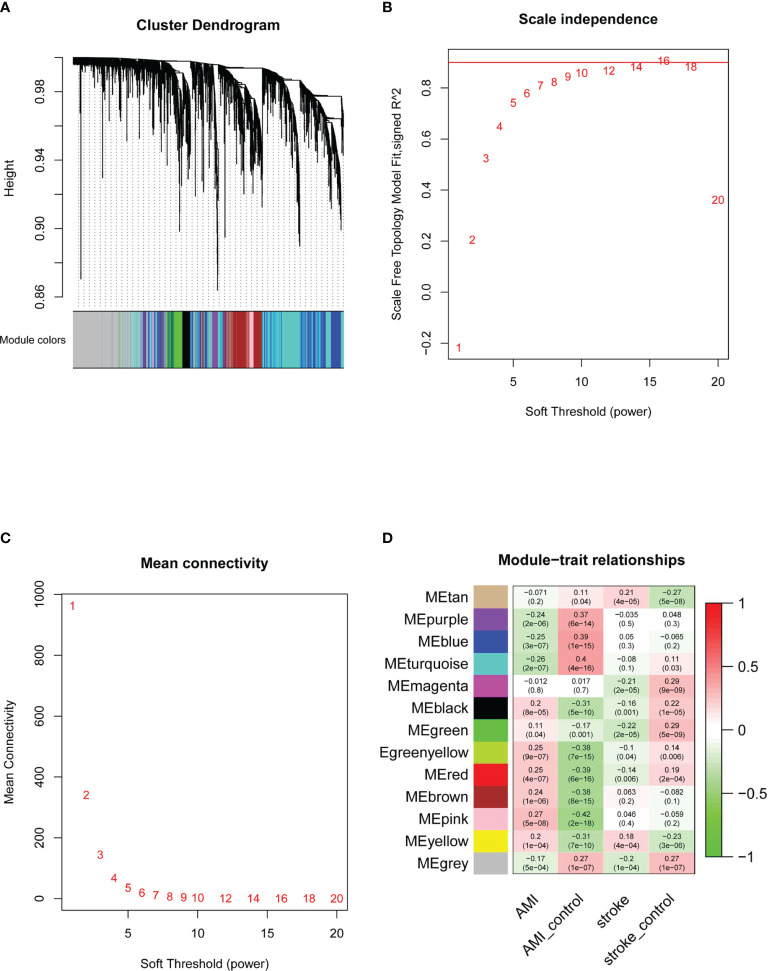
Determination of WGCNA soft-threshold power and identification of modules associated with AMI and IS. **(A)** Dendrogram of DEGs clustered on the basis of the measurement of dissimilarity (1-TOM). The color band indicates the results from automatic single-block analysis. **(B)** Scale-free index analysis for various soft-threshold powers. **(C)** Mean connectivity analysis for various soft-threshold powers. **(D)** Heatmap of the correlation between modules and diseases. TOM, topological overlap matrix; ME, module eigengene. Statistical significance was determined by *t*-test.

Next, we performed WGCNA to identify key modules and hub genes in AMI and IS. After the outliers had been removed, we drew a sample clustering tree (**Figure 3A**) and constructed a scale-free network (**Figures 3B, C**) with a soft threshold of 9 (R^2^ = 0.98). An adjacency matrix was then built and a topological overlap matrix was constructed. A total of 13 modules were identified based on average hierarchical clustering and dynamic tree clipping (**Figure 3D**). The yellow module was strongly and positively related to both AMI and IS, whereas the red module was positively and negatively related to AMI and IS, respectively. Although the pink and green-yellow modules correlated significantly with AMI, they did not correlate significantly with IS. Conversely, the tan and magenta modules correlated significantly with IS but not AMI. Consequently, these six modules were selected as clinically important modules for further analysis.

GO enrichment analyses for cell component (CC), biological process (BP), and molecular function (MF) in these modules were performed using clusterProfiler. In the yellow module, the BP were mainly associated with neutrophil activation, the CC were mainly associated with multiple cellular granules and membranes, and the MF were significantly related to the receptor for advanced glycation end products (RAGE). Together, these results suggest that the yellow module is mainly involved in the activation of inflammation. In the red module, the BP were mainly related to the electron transport chain and ATP metabolic processes, the CC were related to mitochondrial activities, and the MF were associated with electron transfer activity, which demonstrated that the genes of the red module were mainly involved in the regulation of mitochondrial activities. Further information about each module and its hub genes are included in the **Supplementary Data**. Taken together, the findings of these analyses suggest that these six modules are highly associated with clinical situations and that the activation of inflammation and mitochondrial activity could play important roles in the development of AMI and IS.

### Immune Cell Infiltration Analysis

**Figure 4:**
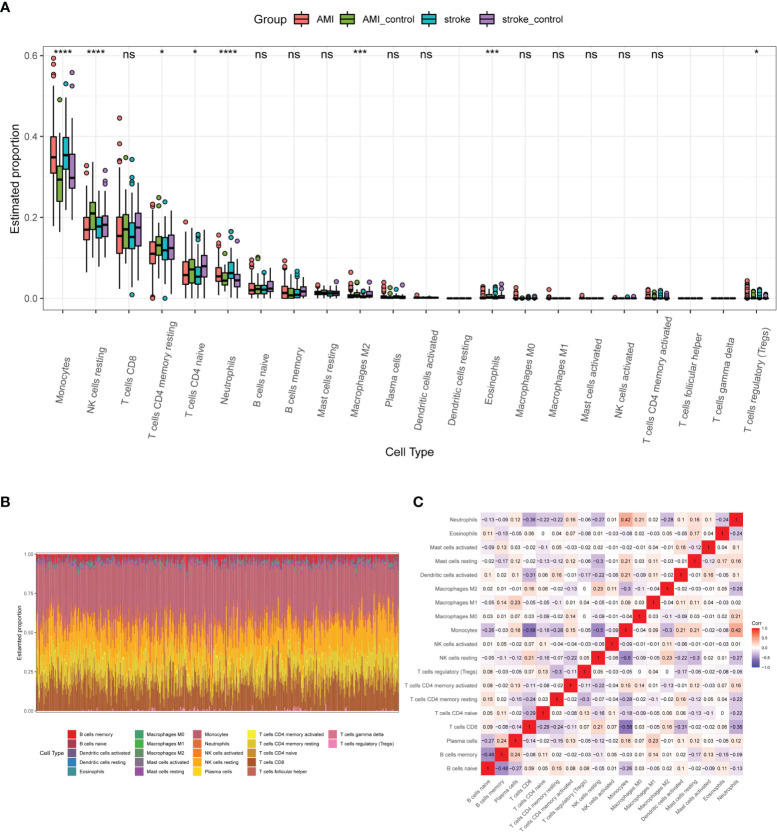
Profiles of immune cell infiltration patterns in the basic datasets. **(A)** Estimated proportions of 22 immune cell types in AMI and IS. Statistical significance was determined by Kruskal test analysis. * *P*-value < 0.05; *** *P*-value < 0.001, **** *P*-value < 0.0001. NS, not significant. **(B)** Heatmap of the immune cell proportions in each sample. **(C)** Correlation heatmap of 19 immune cell types (three were excluded as their proportion was zero).

To further analyze the immune cell infiltration characteristics of AMI and IS, we compared the patterns of immune cell infiltration. Monocytes, NK cells, CD8+T cells, CD4+ memory T cells, CD4+ naïve T cells, neutrophils, and naïve B cells were the main immune cell types in both AMI and IS according to the estimated profiles (**Figure 4A**). However, the distribution of some immune cell subsets with low abundance was not fully revealed due to the limitations of the CIBERSORT algorithm. As reported previously (3, 24, 25), the proportion of monocytes, which play crucial roles in inflammation and tissue repair, increased greatly after both IS and AMI (3, 27, 28). Most immune cell types displayed similar patterns and infiltration extent between AMI and IS, except for NK cells. As shown in **Figure 4A**, despite the estimated proportions of NK cells being lower in both AMI and IS than in the controls, the difference between patients with AMI and the controls is much more significant. The general distribution of immune cells in each sample is shown in **Figure 4B**, in which each immune cell type is represented by a different color and the height represents the percentage of cells in the sample. We also calculated the correlations between these immune cells (**Figure 4C**), finding that monocytes correlated positively with plasma cells, CD4+ T memory cells, dendritic cells, mast cells, and neutrophils, but correlated negatively with naïve B cells, CD8+T cells, NK cells, and M2 macrophages. Furthermore, NK cells correlated positively with CD8+ T cells and M2 macrophages (data not shown). The alterations in NK cell abundance were significantly different between AMI and IS, while other cell types showed no or smaller differences. Consequently, we decided to examine the patterns of NK cell infiltration in AMI and IS in more detail.

### Programmed Cell Death Analysis

**Figure 5:**
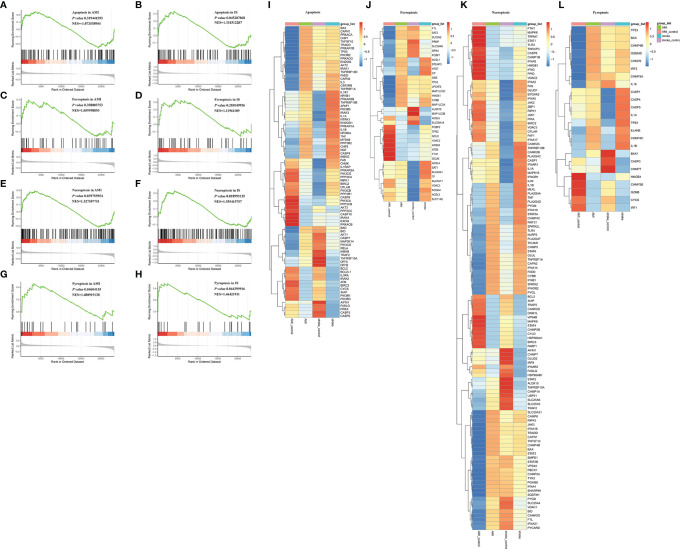
GSEA of apoptosis, necroptosis, ferroptosis, and pyroptosis. **(A)** GSEA of apoptosis in AMI. **(B)** GSEA of apoptosis in IS. **(C)** GSEA of ferroptosis in AMI. **(D)** GSEA of ferroptosis in IS. **(E)** GSEA of necroptosis in AMI. **(F)** GSEA of necroptosis in IS. **(G)** GSEA of pyroptosis in AMI. **(H)** GSEA of pyroptosis in IS. Statistically significant associations were determined by permutation test. **(I)** Heatmaps of DEGs in apoptosis. **(J)** Heatmaps of DEGs in necroptosis. **(K)** Heatmaps of DEGs in ferroptosis. **(L)** Heatmaps of DEGs in pyroptosis.

Besides inflammation, the GO analyses of the DEGs indicated that programmed cell death may also participate in the pathology of AMI and IS (**Supplementary Material Figure 1**), as reported previously (11, 29–31). Therefore, we conducted GSEA to explore the mechanisms underlying cell death in AMI and IS. Due to their prevalence, we decided to focus on apoptosis, ferroptosis, necroptosis, and pyroptosis. As shown in **Figure 5**, apoptosis appeared to play an important role in IS (*p* < 0.05) but not in AMI (**Figures 5A, B**) (*p* > 0.05). Ferroptosis was not involved in either AMI or IS (**Figures 5C, D**) (*p* > 0.05), whereas necroptosis and pyroptosis were significantly involved in both (**Figures 5E**–**H**) (*p* < 0.05).

To further explore the DEGs in each of these forms of cell death, we collected genes related to apoptosis, necroptosis, ferroptosis, and pyroptosis from the Molecular Signatures Database (MSigDB) and conducted GSEA. A total of 88 necroptosis-related genes, 69 apoptosis-related genes, and 17 pyroptosis-related genes were found to be significantly differentially expressed between AMI and IS (**Figures 5I**–**L**). To narrow the scope down, we decided to validate the expression patterns of these genes before further investigating their relationship with NK cells.

### External Validation of Alterations in Apoptosis-Related Genes

**Figure 6:**
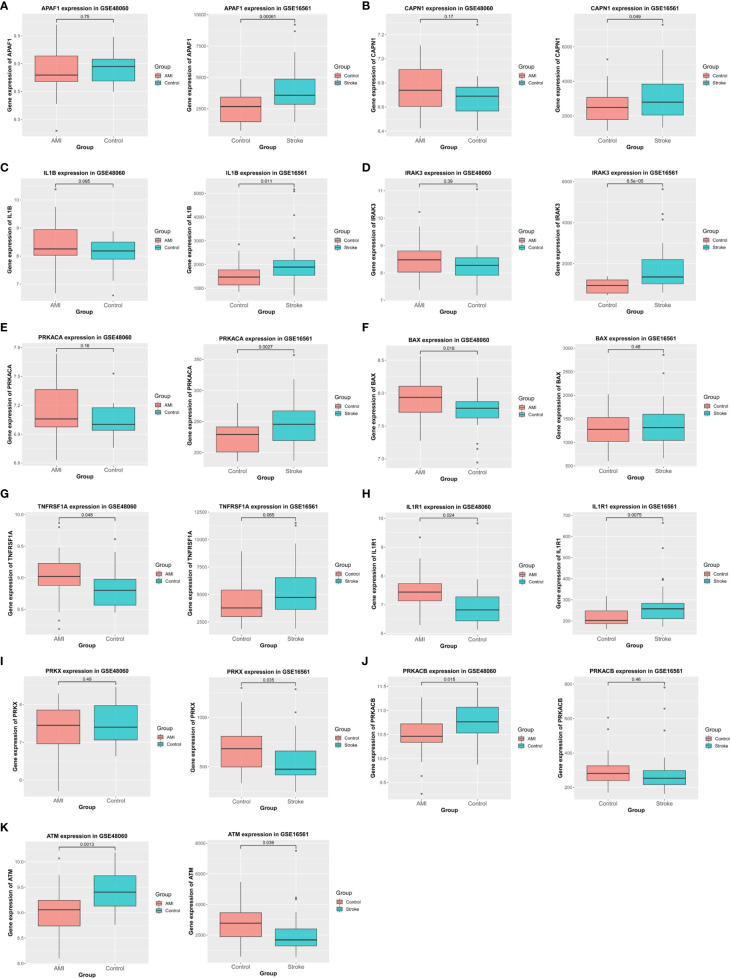
Verification using validation datasets. **(A–K)** Verification of APAF1, CAPN1, IL1B, IRAK3, PRKACA, BAX, TNFRSF1A, IL1R1, PRKX, PRKACB, and ATM expression in the validation cohort. Statistical significance was determined by *t*-test. *P*-values of < 0.05 were considered statistically significant.

To confirm the relationship between these DEGs and apoptosis, we analyzed their expression in the validation datasets and selected genes with the same patterns of alterations in the basic datasets (**Figure 6**). In particular, APAF1, CAPN1, IL1B, IRAK3, and PRKACA were significantly upregulated in IS but not in AMI (**Figures 6A**–**E**), whereas BAX and TNFRSF1A were markedly upregulated in AMI but not in IS (**Figures 6F, G**). Meanwhile, the expression of IL1R1 was elevated in both IS and AMI (**Figure 6H**), and that of PRKX was reduced in IS but not in AMI (**Figure 6I**). Conversely, PRKACB expression was suppressed in AMI but not in IS (**Figure 6J**). Additionally, ATM expression was decreased in both IS and AMI (**Figure 6K**). As such, these genes are likely to be involved in the distinct patterns of NK cell infiltration and apoptosis between AMI and IS.

### Correlation Between Immune Infiltration and Cell Death

**Figure 7:**
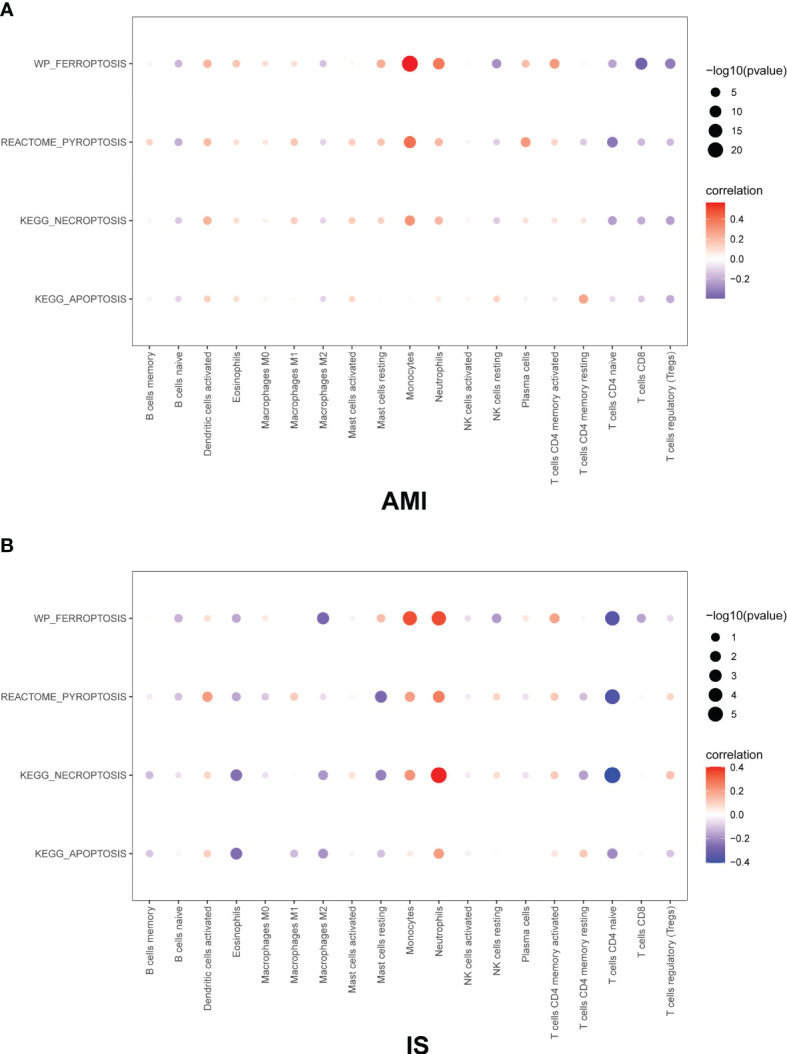
Pearson correlation coefficient between immune cells and cell death in **(A)** AMI and **(B)** IS. The Pearson correlation coefficient was determined by Pearson correlation analysis and the *P*-values were calculated by *t*-test.

Based on our observations, monocytes, NK cells, CD8+ T cells, CD4+ memory T cells, CD4+ naïve T cells, neutrophils, and naïve B cells were the main immune cell types in both AMI and IS. The remaining cell types were estimated to have a low abundance. Then the GEO gene expression matrices were transformed into programmed cell death enrichment matrices by using the GSVA package. We next analyzed the relationship between the patterns of immune cell infiltration, particularly for NK cells, and the types of programmed cell death in AMI and IS (**Figure 7**). Although the proportions of monocytes and neutrophils correlated positively with all four types of cell death, their changing patterns in **Figure 4** show no difference between AMI and IS. Moreover, the proportion of NK cells was positively correlated with apoptosis and negatively correlated with the other three types of cell death. Figures demonstrating the exact correlations and their corresponding *P*-values are included in the **Supplementary Materials (Figures 2, 3)** Thus, the lack of NK cells in AMI appears to be consistent with the deficiency in apoptosis in AMI, meaning that the number of NK cells may be associated with apoptosis in cells within the target organ.

### Correlation Between NK Cells and Apoptosis-Related Genes

**Figure 8:**
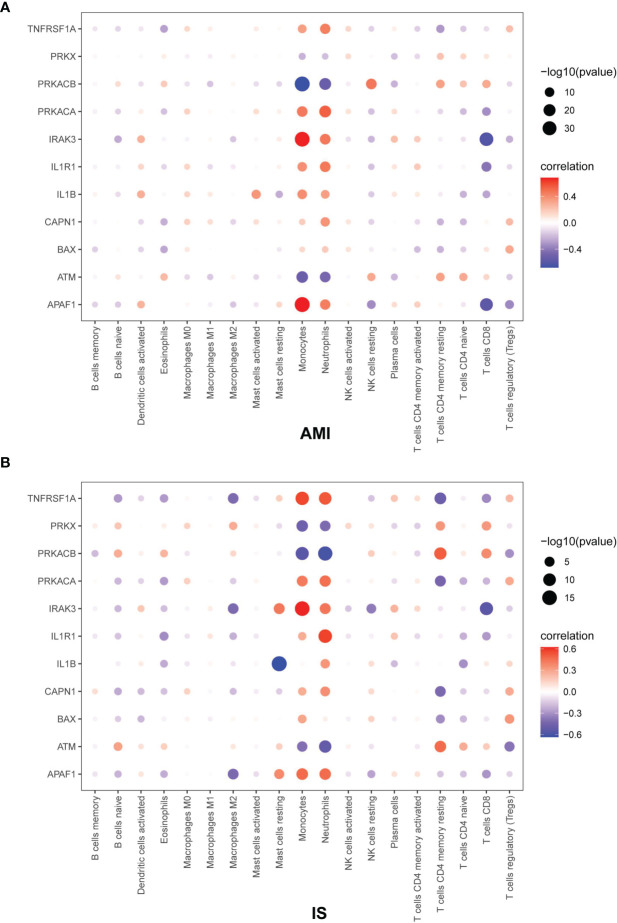
Pearson correlation coefficient between immune cells and apoptosis-related genes in **(A)** AMI and **(B)** IS. The Pearson correlation coefficient was determined by Pearson correlation analysis and the *P*-values were calculated by *t*-test.

To further explore the relationship between NK cells and genes related to cell death, we calculated the Pearson correlation coefficient between immune cells and apoptosis-related genes (**Figure 8**). The values of the Pearson correlation coefficient between each gene and NK cell abundance in AMI and IS as well as the corresponding *P*-values are shown in the **Supplementary Materials (Figures 4–9)**. The following genes were negatively related to NK cell abundance in AMI and IS: TNFRSF1A (AMI: *p* < 0.05, IS: *p* > 0.05), PRKACA (AMI: *p* < 0.05, IS: *p* > 0.05), IRAK3 (AMI: *p* < 0.05, IS: *p* < 0.05), IL1R1 (AMI: *p* < 0.05, IS: *p* > 0.05), and APAF1 (AMI: *p* < 0.05, IS: *p* < 0.05). Conversely, the following genes were positively related to changes in NK cell abundance in both AMI and IS: PRKX (AMI: *p* > 0.05, IS: *p* > 0.05), PRKACB (AMI: *p* < 0.05, IS: *p* > 0.05), IRAK3 (AMI: *p* > 0.05, IS: *p* > 0.05), and ATM (AMI: *p* < 0.05, IS: *p* > 0.05). The expression of several genes also correlated negatively with NK cell abundance in AMI but positively in IS: IL1B (AMI: *p* < 0.05, IS: *p* > 0.05), CAPN1 (AMI: *p* < 0.05, IS: *p* > 0.05), and BAX (AMI: *p* > 0.05, IS: *p* > 0.05). Thus, the correlation between NK cell abundance and the expression of the genes PRKX and BAX was not significant. On the contrary, there was a significant relationship between the expression level of the genes TNFRSF1A, PRKACA, PRKACB, IRAK3, IL1R1, IL1B, CAPN1, ATM, and APAF1 and NK cell abundance. Consequently, these genes could play key roles in the relationship between NK cell abundance and apoptosis in AMI and IS.

## Discussion

Since cardiovascular and cerebrovascular diseases are the leading causes of death worldwide (1), with a high prevalence that increases with age, new approaches to improve cardiac healing are urgently required. Recent studies have highlighted the crucial role that the immune system plays in orchestrating angiogenesis, the resolution of inflammation, the regeneration of injured tissues, and post-infarction tissue remodeling after ischemic injury, including AMI and IS (5, 32). However, inflammation can also facilitate cell death in target organs and infarct expansion, thereby determining the degree of injury and course of the disease. Immune cells can remove necrotic cell debris and produce a milieu that is vital for the reconstruction of the extracellular matrix, neo-vascularization, and subsequent organ recovery. In particular, NK cells possess both direct and indirect cytotoxic effector mechanisms (33). In addition to the exocytosis of lytic granules, activated NK cells can mediate apoptosis by expressing TRAIL and/or Fas ligand (FasL) to engage TRAIL-R1/-R2 or CD95/Fas, respectively, on the surface of diseased cells (33). Moreover, NK cells can interact with inflammatory macrophages *via* the IFN-γ/TNF-α/IL-12 cytokine axis in order to potentiate one another’s activity and increase inflammation in the infarcted zone (6). However, it has also been reported that NK deficiency during AMI is correlated with reduced cardiac cell apoptosis in post-infarcted myocardium (34). In this study, we compared the different expression patterns of genes in AMI and IS. And according to the results of GO and KEGG enrichment, the DEGs were mainly enriched in activities of immune cells and related to the process of cell death. Consequently, we compared the patterns of immune cell infiltration and cell death in patients with AMI or IS. Surprisingly, the alterations in NK cell abundance were significantly different between AMI and IS, while other cell types showed no or smaller differences. Moreover, apoptosis was only statistically significant in IS. After the analyses of the correlation between immune infiltration and cell death as well as the relationship between NK cell abundance and apoptosis related genes. We confirmed for the first time that the proportion of NK cells and the activation of apoptosis are highly correlated. This observation may be partially explained by apoptosis-related genes whose expression clearly correlated with NK cell abundance in AMI and IS.

Tumor necrosis factor (TNF) receptor superfamily member 1A (TNFRSF1A) is a member of the TNF superfamily that encodes the type 1 TNF receptor (TNFR1, TNFRSF1A, p55, CD120A) (35). In this study, we found that it correlated negatively with NK cell abundance in both AMI and IS. Moreover, it was upregulated in these diseases. Heterozygous mutations in TNFRSF1A on chromosome 12p13 have been shown to induce TNF receptor-associated periodic syndrome (TRAPS), a rare dominantly inherited disease characterized by recurrent episodes of fever and generalized/localized inflammation (35, 36). Others have also found an association between the R92Q polymorphism of TNFRSF1A and the development of atherosclerosis as well as inconsistency between the presence of the R92Q in patients with AMI and IS and disease occurrence (37). TNFRSF1A has also been shown to mediate the protective role of cerebellar fastigial nucleus stimulation in reducing the severity of post-stroke depression (38). Together, these findings and researches of others indicate that TNFRSF1A may present genetic changes or polymorphism, thereby leading to different patterns of NK cell infiltration and apoptosis in AMI and IS.

Protein kinase CAMP-activated catalytic subunit alpha (PRKACA) encodes the PKA catalytic subunit alpha (Cα) isoform which has three alternately spliced transcripts in humans: Cα1, Cα2 (Cαs), and Cα3 (39). PRKACB is an important paralog of this gene that encodes Cβ of PKA. The targeted deletion of PRKACA in mice has been found to result in growth retardation in those that survive, while Cα deficiency has been linked to spinal neural tube defects (40, 41). PRKACA has also been reported to be involved in various cancers (42–45). The functions of PRKACA and PRKACB are profoundly associated with those of PKA, a serine/threonine kinase that is responsible for phosphorylating various downstream substrates. Here, we found that PRKACA expression was elevated after IS but not AMI and correlated positively with the abundance of NK cells in IS but negatively with their abundance in AMI. In contrast, PRKACB was downregulated after AMI but not IS and correlated positively with the abundance of NK cells in both AMI and IS. It has been reported that Cα but not Cβ is required for normal immune reactivity, demonstrating that PKA catalytic subunits can exert isoform-specific functions in the same cell (46). However, whether the isoform-specific functions also exist in NK cells or target organ cells during AMI and IS remains unclear.

Interleukin 1 receptor associated kinase 3 (IRAK3) is a member of the IRAK family that encodes IRAK-M, which is only expressed in human monocytes and macrophages in an inducible manner (47–49). IRAK3 has been reported to negatively regulate TLR signaling (50) as well as the alternative NF-κB pathway in a TLR2-specific manner (51). Moreover, silencing IRAK3 impedes cardiac rupture and ventricular remodeling by inactivating the NF-κB signaling pathway (52), while IRAK-M deficiency was shown to increase infarct volume, exacerbate brain edema, elevate the incidence of hemorrhage transformation, and increase inflammatory responses (53). Downregulation of IRAK3 may lead to lesions in the heart and brain caused by hypoxia injury through inflammatory pathways. In our study, we found that IRAK3 was downregulated in both AMI and IS, and correlated negatively with the changes in NK cell abundance.

Interleukin 1 receptor type 1 (IL1R1) belongs to the interleukin 1 receptor family, whose members sense the barrier integrity and fitness of cells (54). IL-1R1 mediates IL-1-dependent activation *via* IL-1α and IL-1β (55). In addition, IL-1R1 has a soluble isoform that inhibits the interaction of soluble IL-1 ligands with IL-1R1 (56), which regulates post-MI remodeling *via* the IL-1R1/cardiac fibroblast signaling axis (57). Brain endothelial and neuronal (cholinergic) IL-1R1 has also been shown to mediate the detrimental effects of IL-1 in the brain during an ischemic stroke (58). In the present study, we showed that IL1R1 was upregulated in both diseases and correlated negatively with NK cell abundance in both AMI and IS. Our findings point to the possibility of different IL1R isoforms being involved in AMI and IS.

Interleukin 1 beta (IL1β) is a central mediator of innate immunity and inflammation (59) that was first identified as being responsible for resistance against microbes (60). IL-1β is produced by various types of cells, including monocytes, macrophages, skin dendritic cells, and brain microglia, in response to Toll-like receptors (TLR), activated complement components, other cytokines (e.g., TNF-α), and IL-1 itself (61). The active form of IL-1β is released into the extracellular space when its precursor is cleaved by caspase-1, which requires proenzyme (procaspase-1) cleavage by the inflammasome. IL1β has been strongly associated with cancers (62, 63) and one IL-1β haplotype has been linked to the risk of stroke in small vessels (64). In this study, we found that IL1β was positively correlated with the abundance of NK cells in IS, but negatively correlated with their abundance in AMI.

Calpain 1 (CAPN1) is a member of the calpain family of at least 15 enzymes (65), among which calpain-1 (μ-calpain) and calpain-2 (m-calpain) are the most ubiquitous in all tissues and organs. Activated calpain-1 may exert neuroprotective effects by cleaving PHLPP1, which activates Akt and leads to neuronal survival (66, 67). In addition, CAPN1 mutations have been reported to induce cerebellar ataxia in some human families and missense mutations in calpain-1 have been found to cause spinocerebellar ataxia in dogs (66, 68, 69). Although some studies have documented that calpains can play crucial roles in ischemia/reperfusion injury in the heart and other organs (70–74), few studies have investigated the role of calpain-1 in AMI and IS. Here, we demonstrated that CAPN1 was upregulated after IS but not in AMI, and correlated positively with the abundance of NK cells in IS but negatively with their abundance in AMI.

Ataxia telangiectasia mutated (ATM) is a protein-coding gene that causes the autosomal recessive disease, ataxia telangiectasia. ATM kinases play key roles in orchestrating signaling cascades that lead to programmed cell death, such as the DNA damage response, DNA repair pathways, and cell cycle checkpoint regulation (75–81). ATM also exerts dual roles in neuronal protection during ischemic preconditioning and the promotion of neuronal death in lethal ischemic injury (82). Similarly, ATM deficiency has been observed to attenuate cardiac dysfunction early post-MI but exacerbate cardiac remodeling during late post-MI by affecting cardiac function, fibrosis, apoptosis, and myocyte hypertrophy (83, 84). Moreover, ATM can mediate the spontaneous regression of Eμ-myc-driven murine B-cell leukemia in a natural killer and T cell-dependent manner (85). In this study, we found that the apoptosis-related gene ATM was significantly downregulated both in AMI and IS. Further analysis revealed that ATM correlated positively with the abundance of NK cells in AMI but not in IS, consistent with our hypothesis that ATM is linked with apoptosis in AMI and IS *via* its effects on NK cell abundance.

Apoptotic peptidase activating factor 1(APAF1) is a key component of the apoptosome which also contains cytochrome c, (d)ATP, and procaspase-9 (86). Under normal conditions, Apaf-1 is stored in an inactive monomeric form in the cytosol. Intracellular stressors such as hypoxia, growth factor deprivation, cell detachment, and stress signals, cause cytochrome c to be released from the mitochondrial intermembrane space into the cytosol where it interacts with and activates Apaf-1, leading to apoptosome formation (86). Apaf-1 has been reported to mediate the beneficial effect of miR-136 during aging (87) and interventions targeting Apaf-1 have demonstrated protective effects in AMI and IS (88–90). Here, we observed a negative correlation between APAF1 expression and changes in NK cell abundance in AMI and IS.

In summary, we hypothesize that apoptosis-related DEGs including TNFRSF1A, PRKACB, PRKACA, IRAK3, IL1R1, IL1B, CAPN1, ATM, and APAF1, together with their correlations with NK cell abundance, likely play important roles in mechanisms underlying the distinct patterns of apoptosis in AMI and IS. However, the function of these genes in NK cells and whether they play different roles in AMI and IS remain to be investigated. Thus, our findings provide potential avenues for the further elucidation of pathogenic mechanisms of ischemic injury in both the heart and brain, as well as promising therapeutic targets for the clinical treatment of AMI and IS.

## Data Availability Statement

Publicly available datasets were analyzed in this study. This data can be found here: http://www.ncbi.nlm.nih.gov/geo.

## Author Contributions

Study design, WY, LL, and YS. Writing—original draft preparation, LF, RT, and XM. Writing—review and editing, CC, YZ, JC, YJS, YL, MZ, and LS. Supervision, WY and LL. All authors contributed to the article and approved the submitted version.

## Funding

This work was supported by the National Natural Science Foundation of China (grants no. 81770345 and 81870266) and the National High Level Talents Special Support Plan.

## Conflict of Interest

The authors declare that the research was conducted in the absence of any commercial or financial relationships that could be construed as a potential conflict of interest.

## Publisher’s Note

All claims expressed in this article are solely those of the authors and do not necessarily represent those of their affiliated organizations, or those of the publisher, the editors and the reviewers. Any product that may be evaluated in this article, or claim that may be made by its manufacturer, is not guaranteed or endorsed by the publisher.

## Supplementary Material

Supplementary Figure 1 GO enrichment analysis in IS. BP, biological process.

Supplementary Figure 2–3 Pearson correlation coefficient between immune cells and cell death in (Left) AMI and (Right) IS. The Pearson correlation coefficient was determined by Pearson correlation analysis and the P-values were calculated by t-test.

Supplementary Figure 4–9 Pearson correlation coefficient between immune cells and apoptosis-related genes in (Left) AMI and (Right) IS. The Pearson correlation coefficient was determined by Pearson correlation analysis and the P-values were calculated by t-test.
