# Supplementary figures and images for: Identification of Genes Linking Natural Killer Cells to Apoptosis in Acute Myocardial Infarction and Ischemic Stroke
Source: Front Immunol. 2022 Apr 1;13:817377. doi: 10.3389/fimmu.2022.817377 (PMC9012496; doi:10.3389/fimmu.2022.817377)

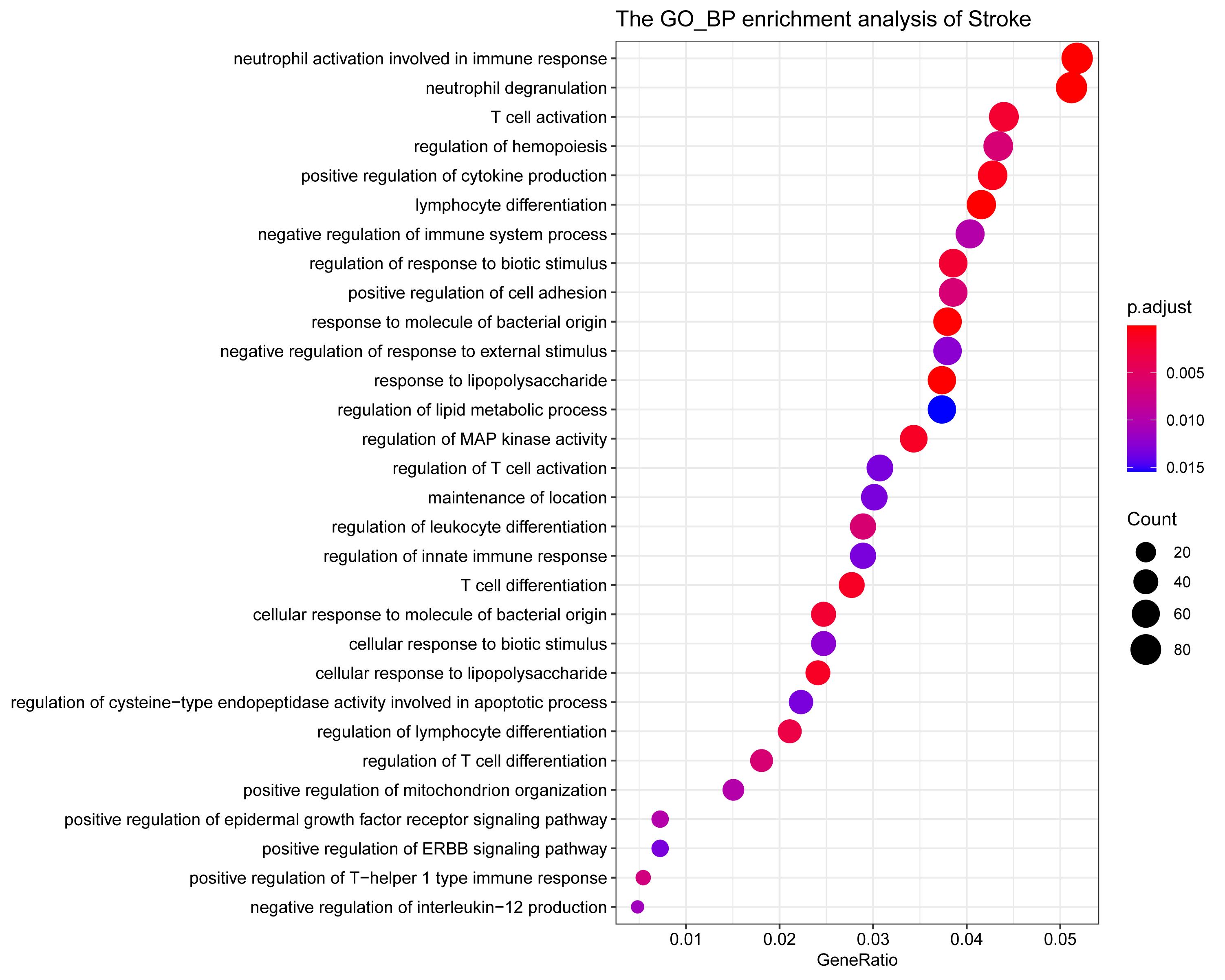

Supplement: Supplementary Figure 1 — GO enrichment analysis in IS. BP, biological process. [file Image_1.tif]

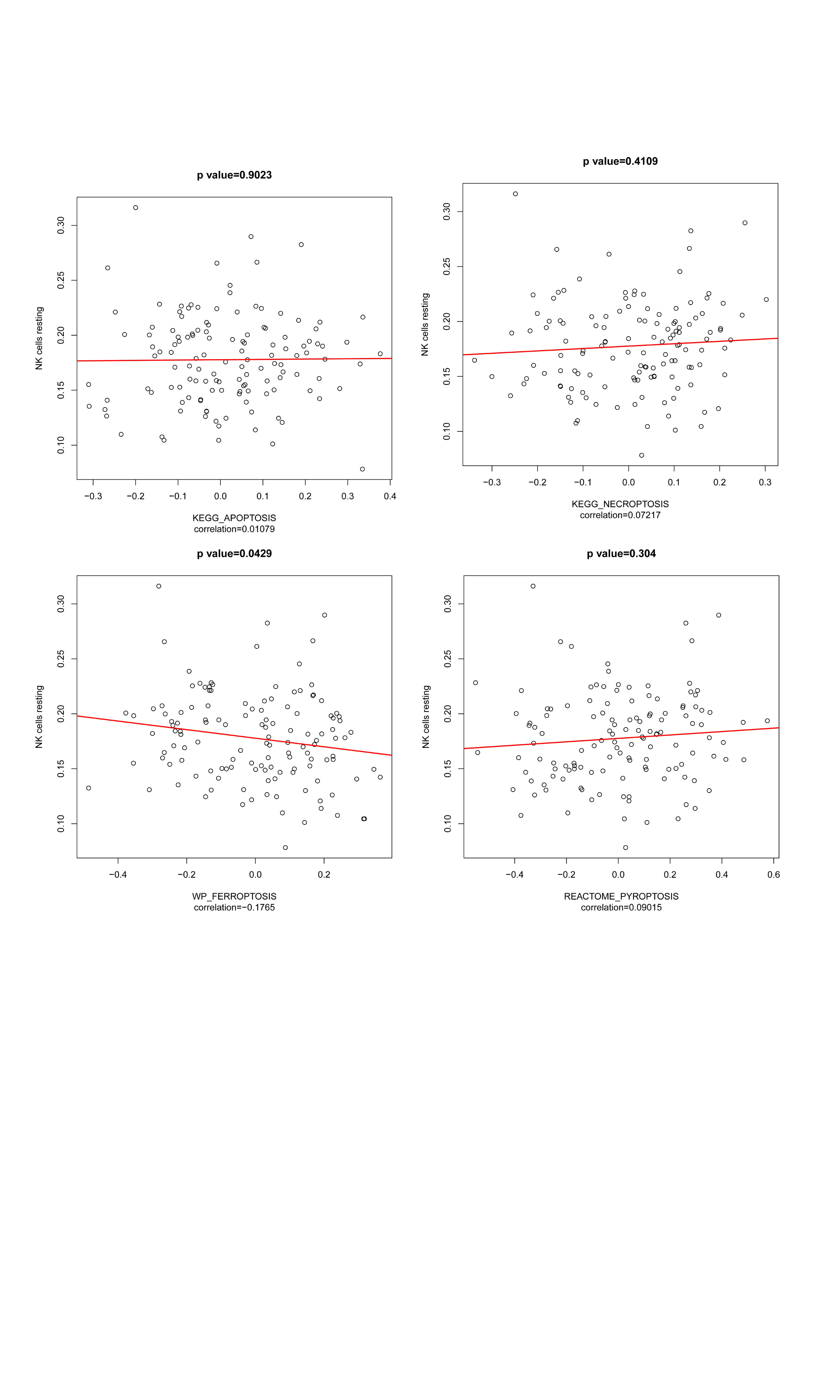

Supplement: Supplementary Figure 2–3 — Pearson correlation coefficient between immune cells and cell death in (Left) AMI and (Right) IS. The Pearson correlation coefficient was determined by Pearson correlation analysis and the P-values were calculated by t-test. [file Image_2.tif]

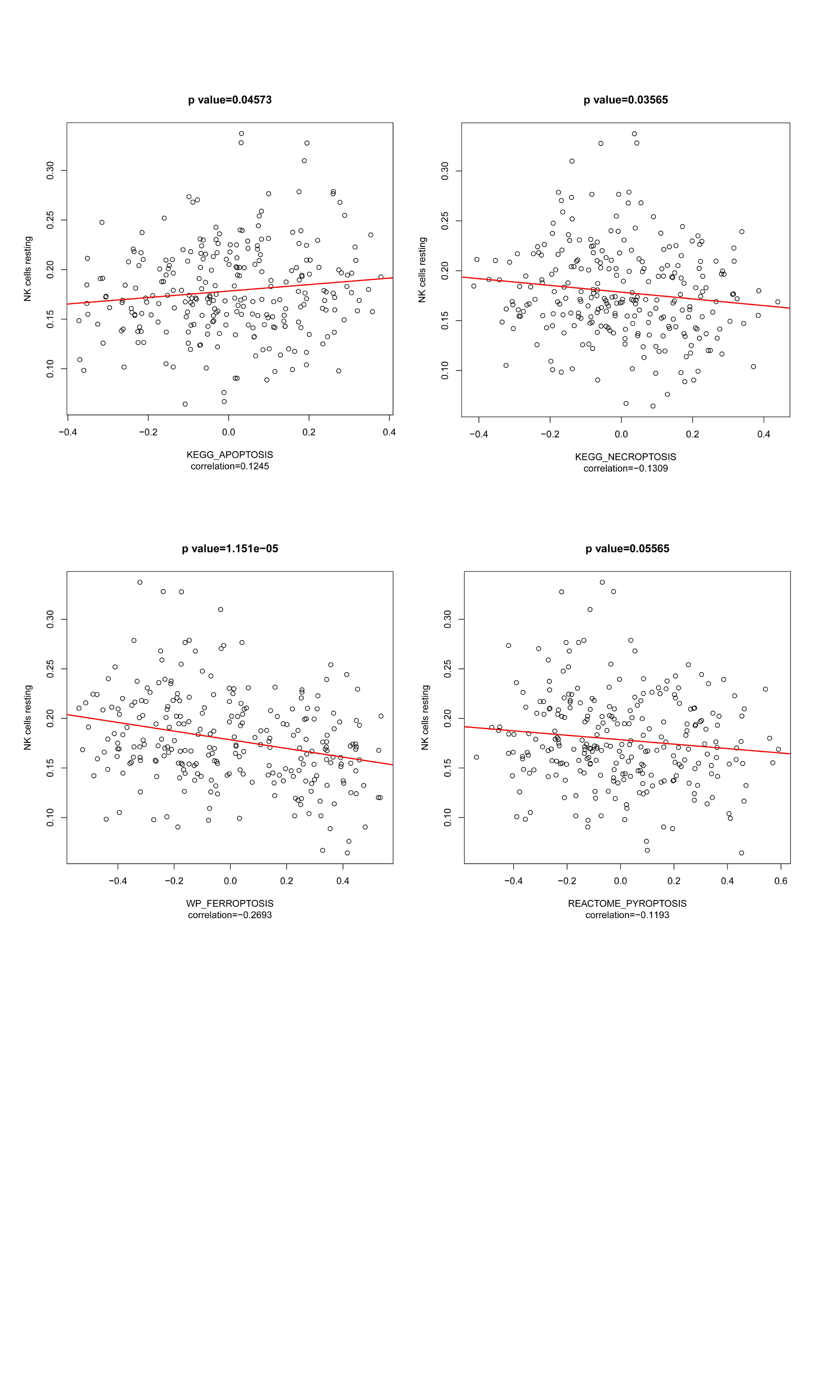

Supplement: Supplementary Figure 4–9 — Pearson correlation coefficient between immune cells and apoptosis-related genes in (Left) AMI and (Right) IS. The Pearson correlation coefficient was determined by Pearson correlation analysis and the P-values were calculated by t-test. [file Image_3.tif]

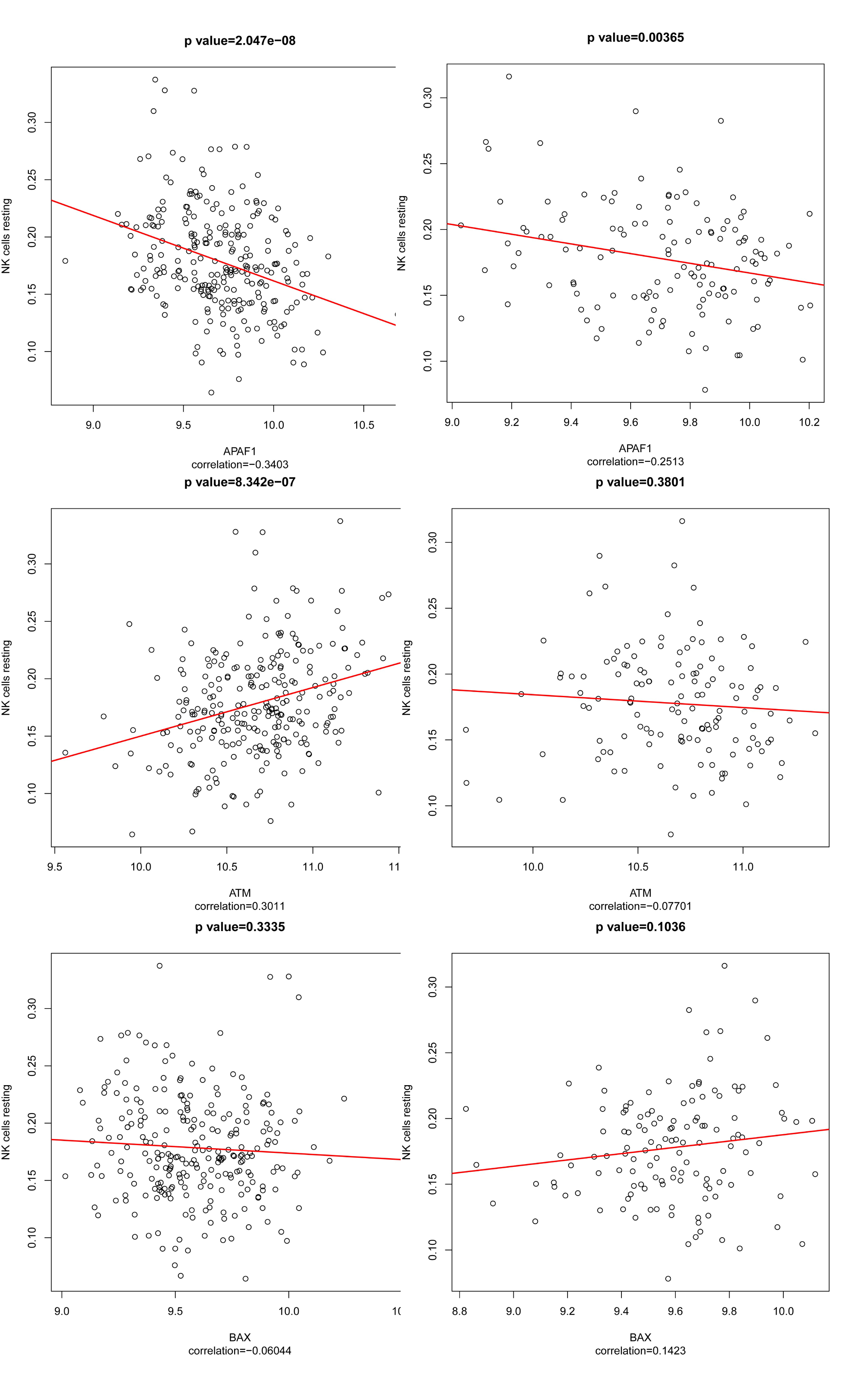

Supplement: Supplementary file 4 [file Image_4.tif]

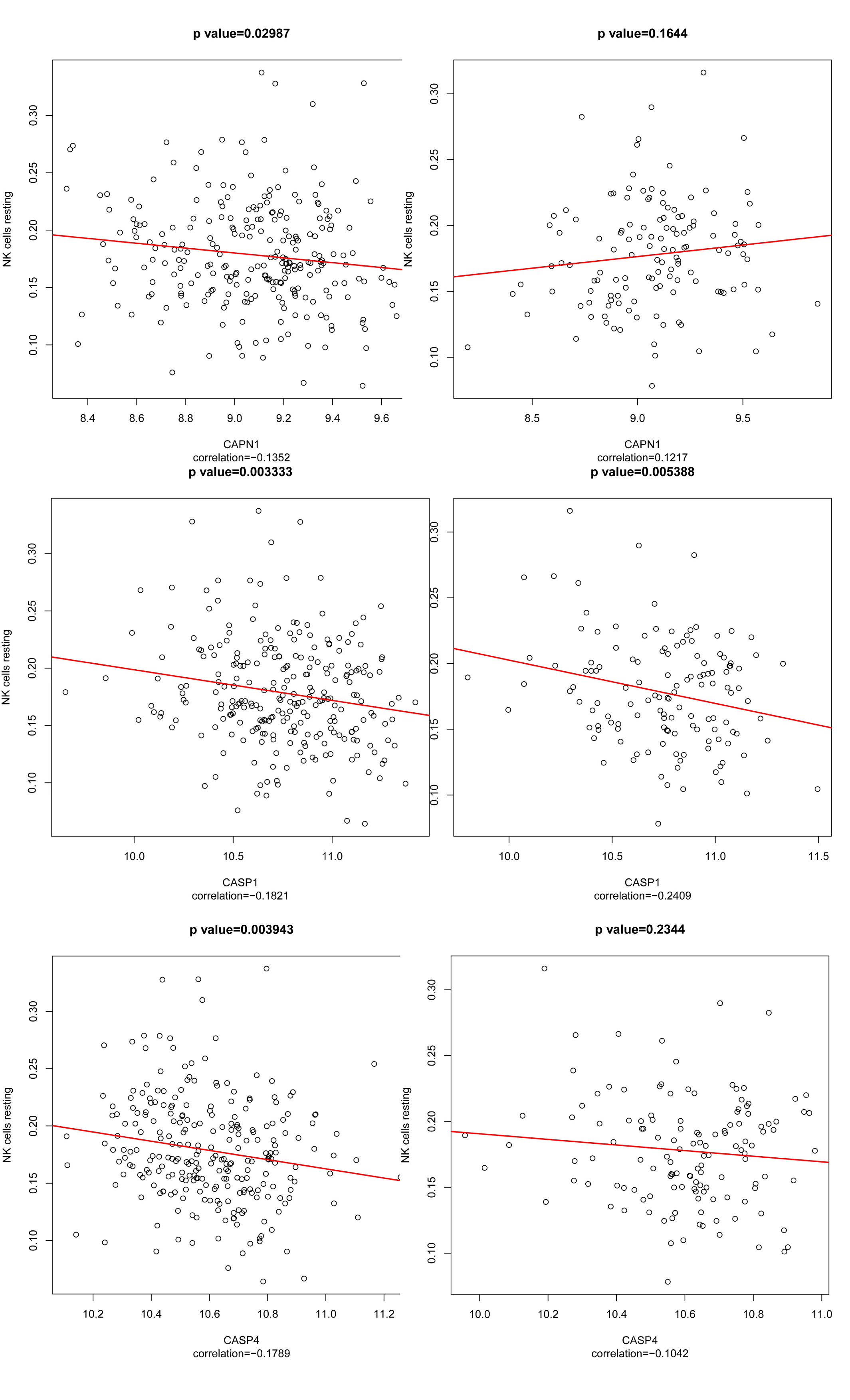

Supplement: Supplementary file 5 [file Image_5.tif]

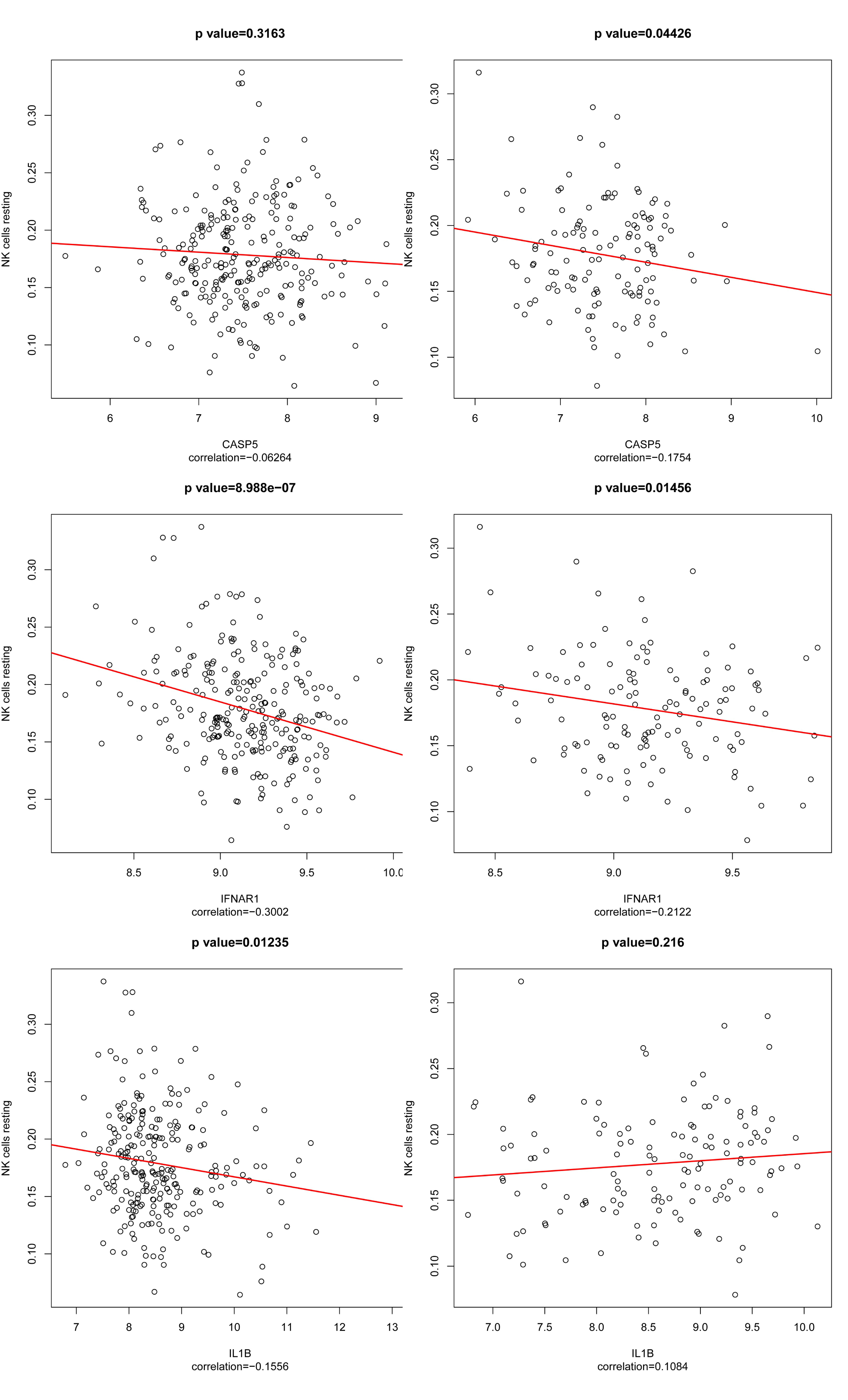

Supplement: Supplementary file 6 [file Image_6.tif]

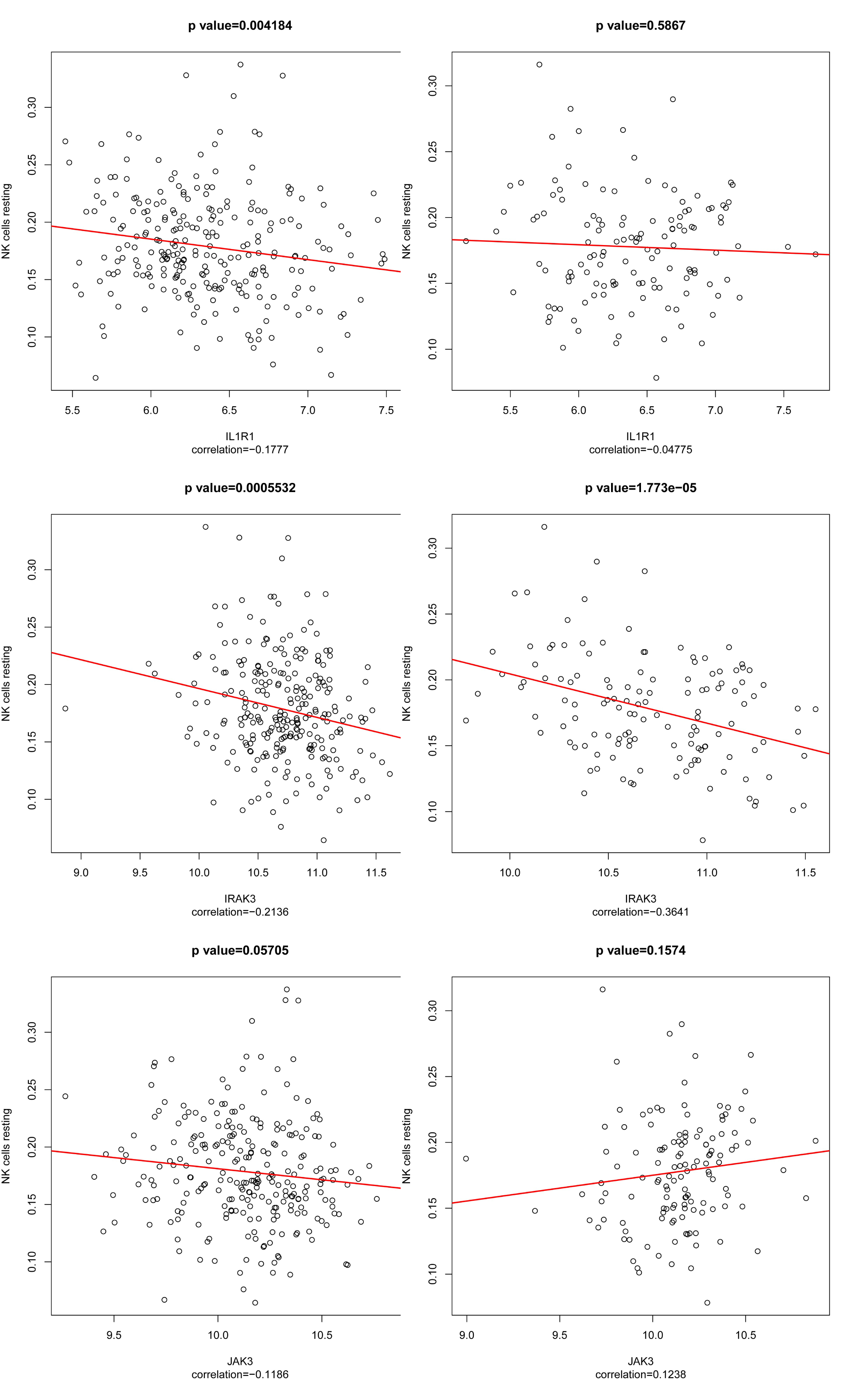

Supplement: Supplementary file 7 [file Image_7.tif]

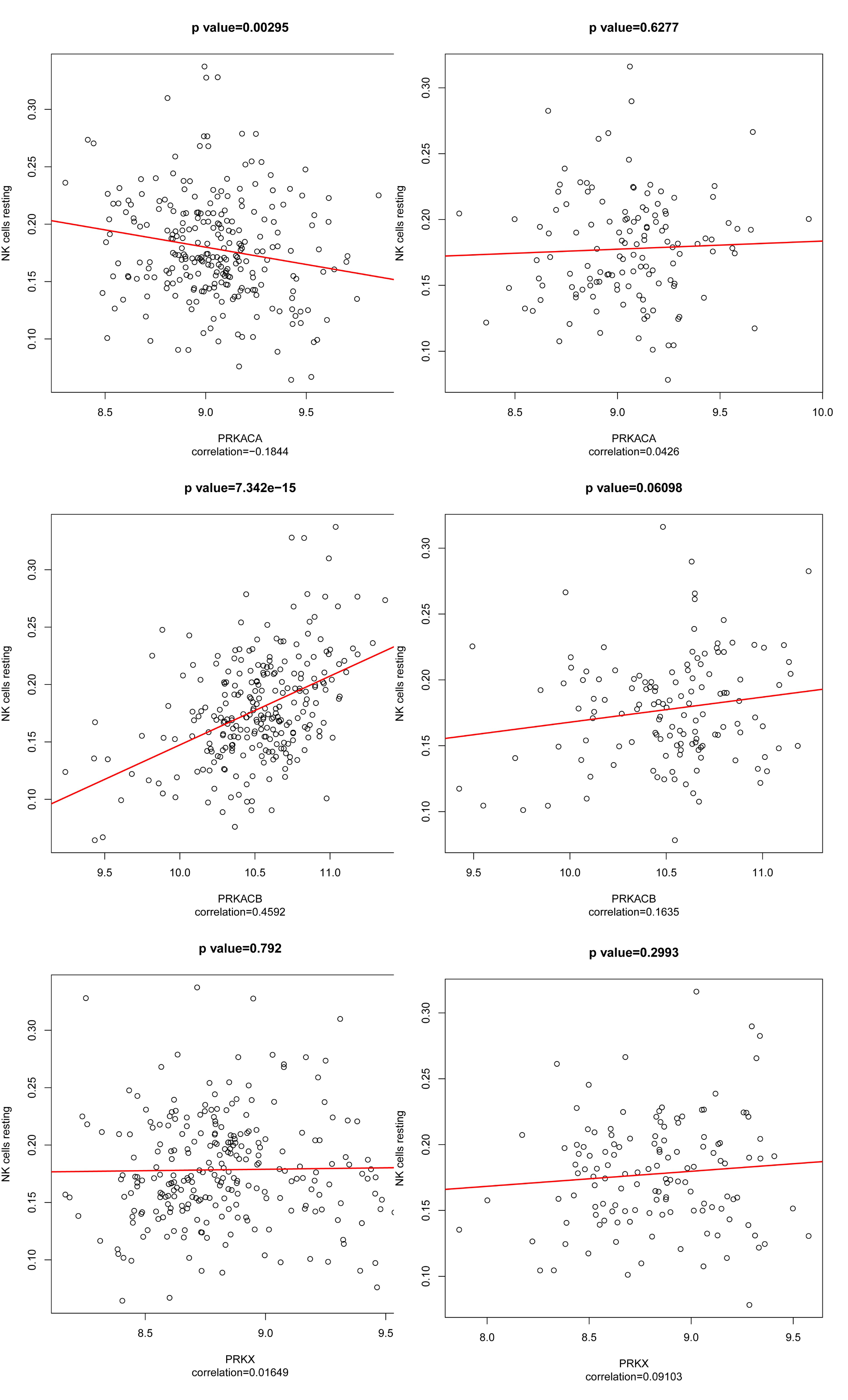

Supplement: Supplementary file 8 [file Image_8.tif]

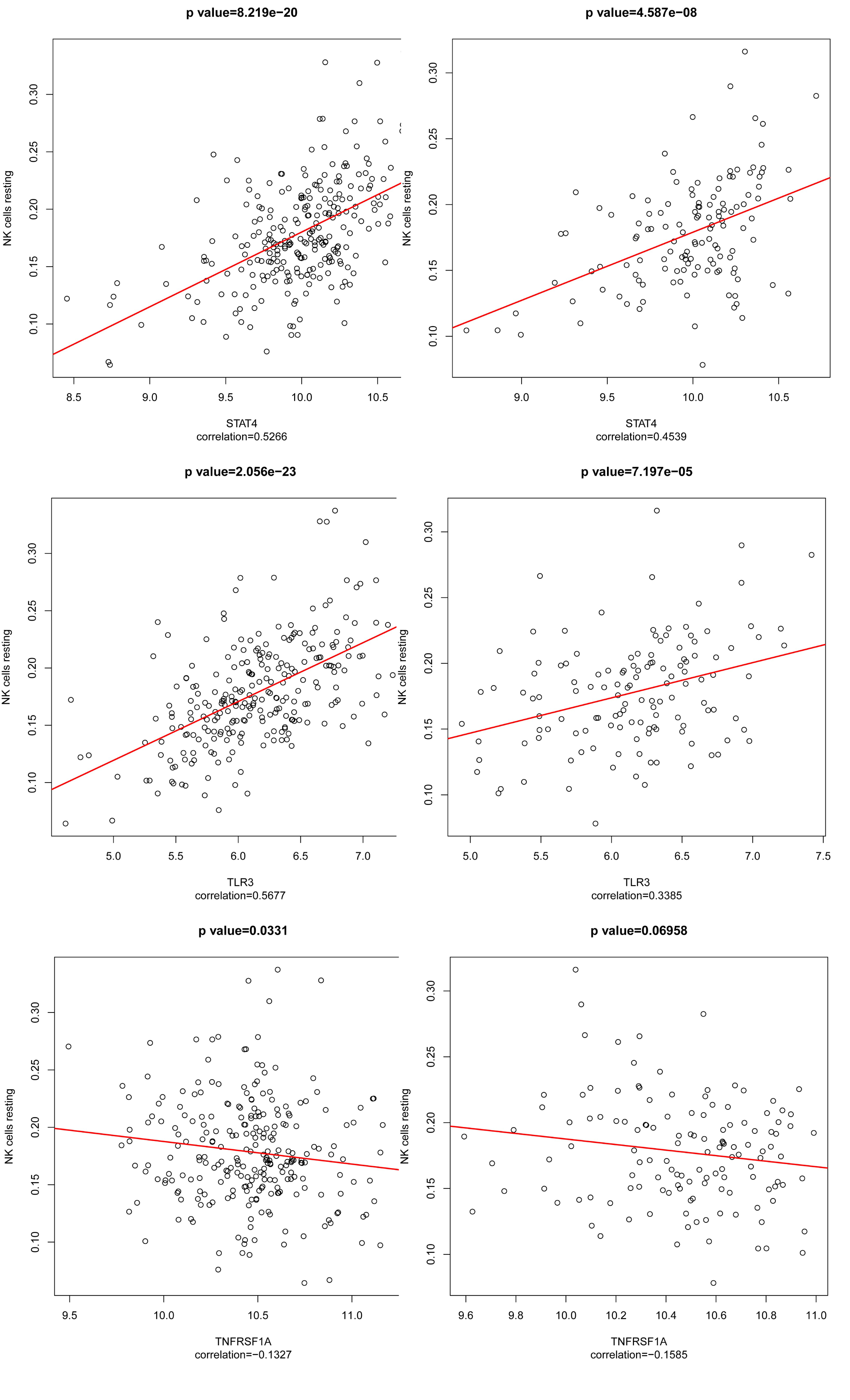

Supplement: Supplementary file 9 [file Image_9.tif]
